# Association between preoperative diagnosis of small airway disease and postoperative pneumonia in resectable esophageal squamous cell carcinoma patients: a retrospective cohort study

**DOI:** 10.7717/peerj.21315

**Published:** 2026-08-26

**Authors:** Sujie Tang, Cheng Shen, Junjie Shi, Mingzhi Zhang, Zhiyun Xu, Qianwei Wang, Dafu Xu, Wenze Tian

**Affiliations:** The Affiliated Huaian No. 1 People’s Hospital of Nanjing Medical University, Huai’an, China

**Keywords:** Esophageal cancer, Esophageal squamous cell carcinoma, Small airway disease, Postoperative pneumonia, The McKeown procedure, Prognosis, Postoperative complications, Perioperative period

## Abstract

**Background:**

Esophageal cancer, specifically esophageal squamous cell carcinoma (ESCC), poses a significant health challenge characterized by a poor prognosis and high mortality rates. Surgical interventions, such as minimally invasive esophagectomy, are the primary treatment approach; however, the occurrence of postoperative complications, particularly pneumonia, presents substantial risks. Small airway disease (SAD) affecting the bronchioles’ small airways is a frequently underestimated respiratory condition with potential implications for the postoperative outcomes of ESCC patients.

**Method:**

From January 2020 to December 2023, the McKeown procedure was exclusively utilized as the treatment modality for resectable esophageal squamous cell carcinoma patients in this retrospective study. Rigorous diagnostic criteria for small airway disease and postoperative pneumonia were employed, followed by detailed statistical analyses using logistic regression models with adjustments for confounding variables. Both multivariate and univariate logistic regression analyses were performed to identify the independent risk factors contributing to postoperative pneumonia incidence.

**Results:**

A total of 293 participants were included in the analysis. Baseline characteristic analysis revealed that individuals with small airway disease exhibited impaired small airway function, lower pre-albumin levels, a higher incidence of pulmonary infection, and longer hospital stays compared to those without SAD. Univariate logistic analysis identified SAD as a strong independent predictor for postoperative pneumonia, with individuals with SAD having a significantly higher risk compared to those without SAD. Multivariate logistic analysis further confirmed this association, demonstrating a robust relationship between SAD and postoperative pneumonia risk even after adjusting for confounding factors.

**Conclusion:**

The preoperative identification of SAD is significantly associated with an increased risk of postoperative pneumonia in resectable ESCC patients, highlighting the importance of addressing small airway disease in perioperative management to enhance clinical outcomes. These findings underscore the significance of recognizing and managing SAD as a potential risk factor in strategies for preventing and managing postoperative pneumonia.

## Introduction

Esophageal cancer places a significant burden on society due to its complexities, poor outlook, and high fatality rates (Waters & Reznik, 2022). In China, it ranks as the sixth most prevalent malignant tumor with the fourth-highest mortality rate, constituting over half of all

In China, it stands as the sixth most common type of cancer with the fourth-highest death rate, making up more than half of all cases (Zhu et al., 2023; Feng et al., 2023). Esophageal squamous cell carcinoma (ESCC) is the most prevalent subtype, representing 95% of diagnosed esophageal cancer instances (Morgan et al., 2022; An, Li & Jia, 2023; Codipilly & Wang, 2022). Treatment options for ESCC mainly consist of radiotherapy, chemotherapy, or esophagectomy, with the goal of achieving complete removal of the cancer (Shen et al., 2022; Puhr, Prager & Ilhan-Mutlu, 2023). Surgery is the main form of treatment, particularly for early-stage esophageal cancer patients (Nakajima & Kato, 2013; Watanabe et al., 2020). Although traditional esophagectomy is a highly invasive procedure, minimally invasive techniques like the McKeown and Ivor Lewis procedures, which involve thoracoscopic/laparoscopic approaches, have gained popularity (Junttila et al., 2023; Sabra et al., 2020; Yun et al., 2020).

Despite a high incidence of postoperative complications nearing 60%, significant challenges persist in managing the complications associated with minimally invasive esophagectomy (MIE) (Duan et al., 2021; Duan et al., 2020; Miao et al., 2024). Postoperative pneumonia is a prevalent issue, impacting 25–30% of cases and often leading to prolonged hospital stays and unfavorable long-term outcomes (Wang et al., 2022b; Shanmugasundaram et al., 2019; Xu et al., 2023b). Recent research has identified risk factors for postoperative pneumonia in patients with esophageal cancer, such as malnutrition, diabetes mellitus, advanced age, increased intraoperative blood loss, thoracotomy, obesity, and recurrent laryngeal nerve palsy (RLNP) (Zhang et al., 2022; Wang et al., 2022a; Maruyama et al., 2022). Enhancing predictive tools for postoperative pneumonia in MIE patients is crucial, given the limited reliability of current indicators as definitive prognostic markers.

Small airway disease (SAD) encompasses various respiratory conditions characterized by abnormalities in the small airways, notably bronchioles with an internal diameter of less than two mm (Papiris et al., 2013; Xu et al., 2023a). Despite its clinical significance, SAD often receives less attention compared to major airway diseases like chronic obstructive pulmonary disease (COPD) or asthma (Yip et al., 2022; Fukuda et al., 2023). However, emerging evidence underscores the critical role of SAD in respiratory health and its potential impact on pulmonary function. Diagnosis of SAD typically involves specialized pulmonary function tests (PFTs), including measurements of maximal expiratory flow rates at different vital capacity percentages (MEF25, MEF50, MEF75/25), providing crucial insights into distal airway airflow limitation and small airway function (Ding et al., 2022). Abnormal MEF25%, MEF50%, or altered MEF75/25 ratios indicate small airway dysfunction, underscoring the significance of early SAD detection and management (Feng-Jia et al., 2018). SAD is associated with diverse respiratory conditions such as bronchiolitis, bronchiolitis obliterans syndrome, and small airway mucus plugs, potentially rendering patients more susceptible to respiratory infections, exacerbations of chronic respiratory diseases, and impaired gas exchange (Barnes et al., 2015; Kligerman et al., 2015). Recent studies suggest a plausible connection between preoperative SAD diagnosis and postoperative pulmonary complications in various surgical populations, as demonstrated in Gong et al. (2024) research linking decreased spirometry reserve ratio and SAD to elevated rates of postoperative pulmonary complications in individuals undergoing laparoscopic gastrectomy for gastric cancer.

Exploring the potential association between preoperative SAD diagnosis and postoperative pneumonia in resectable esophageal squamous cell carcinoma patients undergoing surgery is of paramount interest. Understanding the impact of SAD on postoperative outcomes in this specific patient cohort could significantly influence perioperative management strategies and optimize clinical care protocols. This retrospective cohort study aims to investigate the relationship between SAD and postoperative pneumonia, shedding light on the potential role of small airway disease in the postoperative progression of esophageal squamous cell carcinoma patients.

## Methods

### Study design and participants

A retrospective cohort study was conducted at the Department of Thoracic Surgery, Huai’an No. 1 People’s Hospital, affiliated with Nanjing Medical University, China, covering the period from January 2020 to December 2023. The study focused on patients with resectable esophageal squamous cell carcinoma (ESCC) who underwent thoraco-laparoscopic esophagectomy. Patients meeting the predefined exclusion criteria were excluded from the analysis. The exclusion criteria were as follows: (A) presence of concomitant malignancies; (B) unavailability of preoperative CT imaging data; (C) receipt of neoadjuvant therapy, including chemotherapy, radiotherapy, or chemoradiotherapy; and (D) lack of perioperative clinicopathological data. This exclusion criterion was applied to minimize the potential confounding effects of preoperative oncologic treatment on pulmonary function and postoperative pulmonary outcomes. The inclusion criteria were as follows: (A) patients who underwent preoperative digestive tract endoscopy and received a pathological diagnosis of ESCC; and (B) availability of chest and abdominal CT imaging data obtained within one week before surgery at our hospital. All patients were staged according to the eighth edition of the TNM classification. Ethical approval for the study was obtained from the Medical Ethics Committee of Huai’an First People’s Hospital (Ref. No. KY-2025-246-01). All study procedures adhered to the ethical standards of the Declaration of Helsinki. Given the retrospective nature of the study, the requirement for informed consent was waived by the ethics committee.

### Information on clinical parameters

Clinical parameters retrieved retrospectively from medical records encompassed demographic characteristics, comorbidities, medication details, and laboratory findings. The diagnostic criteria for postoperative pneumonia within a thirty-day post-surgical period were delineated by the following three criteria: (A) The diagnosis required a minimum of two chest radiographs and confirmation of at least one pneumonia diagnosis. (B) Patients must meet at least one of the following conditions: age ≥70 years, peripheral blood white blood cell (WBC) count <4 × 10^9^/L or >12 × 10^9^/L, and fever (>38 °C) accompanied by altered consciousness. (C) Furthermore, patients had to satisfy at least two of the following criteria: presence of purulent sputum or changes in sputum characteristics, increased respiratory secretions, the necessity for augmented sputum clearance efforts, or experiences of dyspnea. Adherence to all three criteria was essential for establishing a definitive diagnosis of postoperative pneumonia within the specified thirty-day postoperative timeframe (Xu et al., 2023b).

### Diagnosis of small airway disease

Preoperative SAD was assessed using pulmonary function indices reflecting distal expiratory airflow limitation, including MEF25, MEF50, and MEF75/25. Because SAD is fundamentally a clinicopathological entity involving the peripheral airways and direct histopathological confirmation is not feasible in routine preoperative evaluation, spirometric parameters were used in this study as clinically applicable surrogate markers of functional small-airway abnormality. According to the predefined diagnostic criteria used in this study, abnormalities in at least two of the above indices were required for classification as SAD (Chan & Lipworth, 2022). This approach is consistent with previous studies indicating that spirometry-defined small airway dysfunction correlates with structural abnormalities of the peripheral airways on imaging and with pathological remodeling of terminal bronchioles reported in the literature (Chen et al., 2024).

### Statistical analysis

Baseline pathological and clinical variables were presented as a range and median for continuous variables, and as proportions and frequencies for categorical variables. The associations between pathological and clinical variables were assessed using chi-square tests. The relationship between postoperative pneumonia and SAD was investigated through univariate and multivariate logistic regression analyses. Three models were utilized for analysis: model 1, adjusting for diabetes mellitus and arrhythmia; model 2, adjusting for diabetes mellitus, arrhythmia, and pre-albumin levels; and model 3, adjusting for diabetes mellitus, arrhythmia, pre-albumin levels, and length of hospital stay. All statistical analyses were conducted using Free Statistics software version 1.9.2 and the R software package (http://www.R-project.org, The R Foundation). Statistical significance was set at *P* < 0.05 to determine significant differences.

## Results

### Baseline characteristics

Table 1 presents the pathological and clinical characteristics of 293 participants enrolled in the work. Results from the study population analysis revealed significant differences in various baseline characteristics based on the presence or absence of SAD. As shown in Table 2, the comparison showed no statistically significant difference in gender distribution between the two groups (*P* = 0.842), with male gender predominant in both groups. Mean age was slightly higher in the SAD group (65.5 ± 6.3 years) compared to the non-SAD group (64.6 ± 6.7 years), although this difference was not statistically significant (*P* = 0.227). Pulmonary function tests indicated comparable values between the two groups for FEV1% (*P* = 0.413) and FEV1/FVC% (*P* = 0.253). However, there were substantial differences in mean values of maximal expiratory flow rates, with MEF25 l/s, MEF50 l/s, and MEF75/25 l/s significantly lower in the SAD group compared to the non-SAD group (all *P* < 0.001), suggesting impaired small airway function in individuals with SAD. Regarding comorbidities, the prevalence of diabetes mellitus, hypertension, smoking, and drinking habits did not show statistically significant differences between the two groups (all *P* > 0.05). Notably, the mean pre-albumin level was significantly lower in the SAD group (258.8 ± 17.6 mg/L) compared to the non-SAD group (337.4 ± 22.1 mg/L) with a *P* value of less than 0.001, indicating potential nutritional implications associated with SAD. Tumor staging assessment displayed comparable distribution across T and N stages in both groups, with no significant differences observed (*P* = 0.743 and *P* = 0.76, respectively). The presence of arrhythmia also showed no significant discrepancy between the groups (*P* = 0.625). However, the incidence of pulmonary infection was notably higher in the SAD group (40.3%) than in the non-SAD group (14.1%), and the difference was statistically significant (*P* < 0.001). Moreover, patients with SAD exhibited a longer mean length of hospital stay (16.7 ± 4.4 days) compared to those without SAD (11.4 ± 1.8 days), with a significant *P* value of less than 0.001, indicating a potential impact of SAD on clinical outcomes and healthcare utilization.

**Table 1:** Clinic and pathological features of patients.

Characteristics	Total (*n* = 293)
Gender, *n* (%)	
Male	208 (71.0)
Female	85 (29.0)
Age (years), Mean ± SD	65.0 ± 6.5
FEV1%, Mean ± SD	92.4 ± 7.9
FEV1/FVC%, Mean ± SD	98.6 ± 5.6
MEF25 (l/s), Mean ± SD	80.2 ± 16.7
MEF50% (l/s), Mean ± SD	81.0 ± 18.8
MEF75/25 (l/s), Mean ± SD	81.8 ± 17.7
Diabetes mellitus, *n* (%)	
No	202 (68.9)
Yes	91 (31.1)
Hypertension, *n* (%)	
No	236 (80.5)
Yes	57 (19.5)
Smoking, *n* (%)	
No	182 (62.1)
Yes	111 (37.9)
Drinking, *n* (%)	
No	192 (65.5)
Yes	101 (34.5)
Pre-albumin (mg/L), Mean ± SD	298.8 ± 44.1
T stage, *n* (%)	
T1	83 (28.3)
T2	87 (29.7)
T3	123 (42.0)
N stage, *n* (%)	
N0	184 (62.8)
N1	64 (21.8)
N2	32 (10.9)
N3	13 ( 4.4)
Arrhythmia, *n* (%)	
No	216 (73.7)
Yes	77 (26.3)
Pulmonary infection, *n* (%)	
No	214 (73.0)
Yes	79 (27.0)
Length of stay (days), Mean ± SD	14.0 ± 4.2

**Table 2:** Baseline characteristics of the study population by small airway disease.

Characteristics	Small airway disease		*P* value
	No (*n* = 149)	Yes (*n* = 144)	
Gender, *n* (%)			0.842
Male	105 (70.5)	103 (71.5)	
Female	44 (29.5)	41 (28.5)	
Age (years), Mean ± SD	64.6 ± 6.7	65.5 ± 6.3	0.227
FEV1%, Mean ± SD	92.8 ± 9.0	92.1 ± 6.5	0.413
FEV1/FVC%, Mean ± SD	98.9 ± 5.7	98.2 ± 5.4	0.253
MEF25%, Mean ± SD	93.6 ± 7.9	66.3 ± 11.2	<0.001
MEF50%, Mean ± SD	96.7 ± 9.6	64.8 ± 10.5	<0.001
MEF75/25%, Mean ± SD	96.0 ± 6.8	67.0 ± 12.7	<0.001
Diabetes mellitus, *n* (%)			0.492
No	100 (67.1)	102 (70.8)	
Yes	49 (32.9)	42 (29.2)	
Hypertension, *n* (%)			0.378
No	123 (82.6)	113 (78.5)	
Yes	26 (17.4)	31 (21.5)	
Smoking, *n* (%)			0.708
No	91 (61.1)	91 (63.2)	
Yes	58 (38.9)	53 (36.8)	
Drinking, *n* (%)			0.166
No	92 (61.7)	100 (69.4)	
Yes	57 (38.3)	44 (30.6)	
Pre-albumin (mg/L), Mean ± SD	337.4 ± 22.1	258.8 ± 17.6	<0.001
T stage, *n* (%)			0.743
T1	40 (26.8)	43 (29.9)	
T2	47 (31.5)	40 (27.8)	
T3	62 (41.6)	61 (42.4)	
N stage, *n* (%)			0.76
N0	92 (61.7)	92 (63.9)	
N1	31 (20.8)	33 (22.9)	
N2	18 (12.1)	14 (9.7)	
N3	8 (5.4)	5 (3.5)	
Arrhythmia, *n* (%)			0.625
No	108 (72.5)	108 (75)	
Yes	41 (27.5)	36 (25)	
Pulmonary infection, *n* (%)			<0.001
No	128 (85.9)	86 (59.7)	
Yes	21 (14.1)	58 (40.3)	
Length of stay (days), Mean ± SD	11.4 ± 1.8	16.7 ± 4.4	<0.001

### Univariate logistic analysis of postoperative pneumonia

As shown in Table 3, the univariate logistic analysis revealed significant predictors of postoperative pneumonia in the study population. Gender, age, diabetes mellitus, hypertension, smoking, and drinking habits did not show statistically significant associations with postoperative pneumonia (all *P* > 0.05). However, pre-albumin levels demonstrated a significant inverse correlation with the development of postoperative pneumonia (OR 0.98, 95% CI [0.98–0.99], *P* < 0.001), indicating a potential protective effect. Tumor staging (T stage and N stage) did not exhibit significant associations with postoperative pneumonia risk in the univariate analysis (all *P* > 0.05). While the presence of arrhythmia showed a trend towards increased risk (OR 1.7, 95% CI [0.97–2.99], *P* = 0.064), it did not reach statistical significance. Notably, the length of hospital stay was identified as a significant predictor for postoperative pneumonia, with each additional day associated with a 16% increased risk (OR 1.16, 95% CI [1.09–1.24], *P* < 0.001), highlighting the importance of considering the length of hospitalization in postoperative pneumonia prevention strategies. Importantly, the presence of small airway disease emerged as a strong independent predictor of postoperative pneumonia, with individuals having SAD exhibiting a substantially higher risk (OR 4.11, 95% CI [2.33–7.26], *P* < 0.001) compared to those without SAD.

**Table 3:** Results of univariate logistic analysis and predictors of postoperative pneumonia.

Clinical variables	Postoperative pneumonia	
	OR (95% CI)	*P* value
Gender		
Male	Ref	
Female	1.19 (0.68∼2.08)	0.546
Age (years)	0.99 (0.95∼1.03)	0.629
Diabetes mellitus		
No	Ref	
Yes	0.62 (0.35∼1.13)	0.117
Hypertension		
No	Ref	
Yes	0.96 (0.5∼1.85)	0.902
Smoking		
No	Ref	
Yes	1.01 (0.59∼1.71)	0.984
Drinking		
No	Ref	
Yes	1.06 (0.62∼1.82)	0.832
Pre-albumin (mg/L)	0.98 (0.98∼0.99)	<0.001
T stage		
T1	Ref	
T2	0.88 (0.45∼1.73)	0.718
T3	0.86 (0.46∼1.61)	0.647
N stage		
N0	Ref	
N1	0.97 (0.51∼1.82)	0.918
N2	0.35 (0.12∼1.06)	0.062
N3	1.1 (0.32∼3.72)	0.88
Arrhythmia		
No	Ref	
Yes	1.7 (0.97∼2.99)	0.064
Length of stay (days)	1.16 (1.09∼1.24)	<0.001
Small airway disease		
No	Ref	
Yes	4.11 (2.33∼7.26)	<0.001

### Multivariate logistic analysis of postoperative pneumonia

As shown in Table 4, the multivariable-adjusted analysis demonstrated a robust association between SAD and postoperative pneumonia risk. In the crude model, individuals with SAD had a significantly higher odds of developing postoperative pneumonia compared to those without SAD (OR 4.11, 95% CI [2.33–7.26], *P* < 0.001). After adjusting for confounding factors, the association remained strong and statistically significant in all subsequent models. In Model 1, which adjusted for diabetes mellitus and arrhythmia, the OR for postoperative pneumonia in individuals with SAD was 4.26 (95% CI [2.39–7.6], *P* < 0.001), indicating an independent relationship between SAD and postoperative pneumonia beyond the influence of these comorbidities. Further adjustment in Model 2 for pre-albumin levels maintained a significant association, with individuals having SAD showing an OR of 3.14 (95% CI [2.31–4.15], *P* < 0.01) for postoperative pneumonia compared to those without SAD. This suggests that the impact of SAD on postoperative pneumonia risk is partially mediated by pre-albumin levels. In the fully adjusted Model 3, which also accounted for the length of hospital stay, the association between SAD and postoperative pneumonia persisted, with an OR of 3.04 (95% CI [2.29–4.11], *P* < 0.01), indicating that SAD remains an independent predictor of postoperative pneumonia risk even after considering the length of hospitalization. These findings highlight the significant and consistent relationship between SAD and postoperative pneumonia, underscoring the importance of addressing small airway disease as a potential risk factor in the prevention and management of postoperative complications in clinical practice.

**Table 4:** Multivariable-adjust ORs and 95% CI of the small airway disease associated with postoperative pneumonia.

SAD	Crude model		Model 1		Model 2		Model 3	
	OR (95% CI)	*P* value	OR (95% CI)	*P* value	OR (95% CI)	*P* value	OR (95% CI)	*P* value
No	Ref		Ref		Ref		Ref	
Yes	4.11 (2.33–7.26)	<0.001	4.26 (2.39∼7.6)	<0.001	3.14 (2.31∼4.15)	<0.01	3.04 (2.29∼4.11)	<0.01

**Notes.**

Model 1 adjust for diabetes mellitus and arrhythmia. Model 2 adjust for Model 1 and pre-albumin. Model 3 adjust for Model 2 and length of stay.

## Discussion

In this retrospective cohort study of patients with resectable ESCC undergoing thoraco-laparoscopic esophagectomy, preoperative SAD was significantly associated with an increased risk of postoperative pneumonia. Importantly, this association remained robust after multivariable adjustment, suggesting that SAD is not merely a coincidental spirometric finding but may represent a clinically meaningful marker of pulmonary vulnerability in the perioperative setting. Our findings extend the current understanding of postoperative pulmonary risk in esophagectomy by highlighting the potential contribution of distal airway dysfunction in addition to more established factors such as nutritional status and comorbidities. From a clinical perspective, these results support the value of identifying SAD before surgery as part of a more comprehensive perioperative respiratory risk assessment.

Esophagectomy for ESCC remains a highly invasive procedure despite advances in minimally invasive techniques, and postoperative pulmonary complications continue to be among the most important determinants of short-term recovery (He et al., 2021). Within this clinical context, our findings suggest that preoperative SAD may represent an additional and previously underappreciated risk factor for postoperative pneumonia. Rather than viewing SAD as an isolated pulmonary function abnormality, it may be more appropriate to interpret it as a marker of impaired distal airway reserve that becomes clinically relevant when patients are exposed to the physiological stress of major thoracoabdominal surgery.

From a mechanistic perspective, SAD should not be viewed merely as an abnormality of pulmonary function, but rather as a biologically active disorder of the peripheral airways (Chen et al., 2023). Small airways are generally defined as bronchioles with an internal diameter of less than two mm, and pathological studies have shown that these distal airways are a major site of inflammatory and structural injury (Yang et al., 2024). In particular, studies in COPD-related small airway disease have demonstrated chronic inflammatory cell infiltration, airway wall thickening, luminal narrowing, mucus plugging, and remodeling or even loss of terminal bronchioles. These observations support the concept that SAD reflects underlying peripheral airway inflammation and structural vulnerability rather than an isolated functional disturbance (Nakajima et al., 2018).

This pathophysiological framework provides a plausible explanation for the increased postoperative pneumonia risk observed in our study. Inflammation-related narrowing and remodeling of distal airways can promote ventilation heterogeneity, gas trapping, impaired mucociliary clearance, and retention of airway secretions (Sturton, Persson & Barnes, 2008). In the perioperative setting of esophagectomy, these abnormalities may be further aggravated by surgical stress, postoperative pain, transient immunosuppression, reduced cough efficiency, impaired mobilization, and difficulty in sputum clearance (Hogg & Timens, 2009). Consequently, patients with preoperative SAD may enter surgery with a less resilient distal airway environment that is more susceptible to postoperative pulmonary infection. This interpretation is in line with our finding that SAD remained independently associated with postoperative pneumonia after adjustment for major confounders (Brandsma et al., 2020).

Another important implication of our findings is the potential predictive value of preoperative SAD assessment. In routine clinical practice, direct histopathological confirmation of peripheral airway disease is not feasible, whereas spirometric indices such as MEF25, MEF50, and MEF75/25 are readily obtainable before surgery (Jerkic et al., 2020; Hogg, 2004; Carroll et al., 1996). Although these indices do not provide morphological confirmation, recent imaging-based studies have shown that spirometry-defined small airway dysfunction correlates with structural abnormalities of the peripheral airways, including thickened airway walls, narrowed lumens, and reduced airway branch counts on High-Resolution Computed Tomography (HRCT) (Higham et al., 2019). Therefore, preoperative identification of SAD may serve as a practical surrogate marker of distal airway vulnerability and may complement conventional spirometric variables in perioperative risk stratification (Zhang et al., 2023).

Future prospective studies are warranted to validate the predictive role of preoperative SAD in larger and multicenter ESCC cohorts (Zhang et al., 2024). It would also be valuable to investigate whether targeted perioperative interventions, such as preoperative respiratory rehabilitation, smoking cessation, airway clearance training, optimization of secretion management, and intensified postoperative pulmonary surveillance, can reduce pneumonia risk in patients identified as having SAD before surgery (Scelo et al., 2024).

This study has several limitations. First, it was a retrospective single-center study and is therefore subject to inherent selection bias. Second, all included cases were ESCC, which may limit the generalizability of our findings to other histological subtypes of esophageal cancer. Third, the diagnosis of SAD in this study was based on pulmonary function indices rather than direct histopathological or imaging confirmation of peripheral airway lesions. Although this reflects real-world preoperative practice and is supported by prior structure-function studies, some degree of disease misclassification remains possible. Finally, because patients who had received neoadjuvant therapy were excluded, our findings should be interpreted specifically in the context of surgery-first patients.

## Conclusions

Preoperative SAD was significantly associated with postoperative pneumonia in patients with resectable ESCC undergoing thoraco-laparoscopic esophagectomy. These findings suggest that SAD may represent a clinically relevant marker of distal airway vulnerability in the perioperative setting. Recognition of SAD before surgery may help improve risk stratification and support individualized perioperative preventive strategies aimed at reducing postoperative pulmonary complications.

## Supplemental Information

10.7717/peerj.21315/supp-1 Supplemental Information 1 SAD data

10.7717/peerj.21315/supp-2 Supplemental Information 2 Codebook

10.7717/peerj.21315/supp-3 Supplemental Information 3 STROBE checklist

10.7717/peerj.21315/supp-4 Supplemental Information 4 STROBE table

## Additional Information and Declarations

### Data Availability

The following information was supplied regarding data availability: The datasets and codebook are available in the Supplemental Files.
